# An AI-driven conceptual framework for detecting fake news and deepfake content: a systematic review

**DOI:** 10.3389/frai.2026.1737790

**Published:** 2026-03-02

**Authors:** Bravlyn VC. Moyo, Tite Tuyikeze, Fezile Matsebula, Ibidun C. Obagbuwa

**Affiliations:** 1 Department of Computer Science & Information Technology, Faculty of Natural and Applied Sciences, Sol Plaatje University, Kimberley, South Africa; 2 Department of Mathematical Sciences and Computing, Faculty of Natural Sciences, Walter Sisulu University, Mthatha, South Africa

**Keywords:** deepfakes, explainable AI (XAI), generative artificial intelligence, media trust, misinformation, multimodal detection

## Abstract

The rapid advancement of generative artificial intelligence (AI) has enabled the creation of highly realistic synthetic media, commonly referred to as deepfakes, which are increasingly multimodal and difficult to detect. While these technologies offer creative and commercial potential, they also pose critical challenges related to misinformation, media trust, and societal harm. Despite the growing body of research, existing reviews remain fragmented, often separating technical detection advances from social and governance considerations. This study addresses this gap through a systematic review conducted in accordance with PRISMA guidelines across IEEE Xplore, Scopus, ACM Digital Library, and Web of Science. From an initial set of 120 database records, complemented by citation chaining, 34 studies published between 2014 and 2025 were included for analysis. Eighteen studies focused on deepfake generation and detection models, eight examined social and behavioural implications, and eight addressed ethical and regulatory frameworks. Thematic synthesis reveals a clear methodological shift from convolutional neural networks toward transformer- and CLIP-based architectures, alongside the emergence of large-scale benchmark datasets. However, persistent challenges remain in multimodal detection, cross-dataset generalization, explainability–robustness trade-offs, and the translation of governance principles into deployable systems. This review contributes an integrated conceptual framework that operationally connects detection technologies, explainable AI (XAI), and governance mechanisms through explicit feedback loops. Future research directions emphasize robust multimodal benchmarks, retrieval-augmented detection systems, and interdisciplinary approaches that align technical innovation with ethical and policy safeguards.

## 1 Introduction

Generative AI has emerged as a transformative force in digital content creation. Among its most striking applications are deepfakes synthetically generated or manipulated videos, images, and audio that can convincingly imitate real individuals. Initially conceived as technical demonstrations, deepfakes have evolved into powerful tools with dual-use potential, supporting both creative innovation and malicious activities such as non-consensual sexual content, political misinformation, and reputational harm. Recent studies have investigated deepfake creation and identification in visual, audio, and multimodal domains, as well as the social and cognitive impacts of misinformation (Chuk-Ke and Dong, 2024; Donahue et al., 2019; Gowrisankar and Thing, 2024; Green and Swets, 1966; Kumar et al., 2022; Loth et al., 2024; Nguyen et al., 2020, 2022; Pearson and Zinets, 2022; Rossler et al., 2019; Siarohin et al., 2019; Shu et al., 2020; Sweller, 1988; Zhang et al., 2024).

The implications of deepfakes extend well beyond technical domains, intersecting with media trust, democratic governance, and legal accountability. Social science research has examined misinformation dynamics (Zhou et al., 2021; Idiongo, 2024), while policymakers have begun formulating governance frameworks such as the EU Digital Services Act (European Parliament and Council of the European Union (EU DSA), 2022) and the EU AI Act (European Parliament and Council of the European Union (EU AI Act), 2024). In parallel, computer vision research has advanced rapidly, developing datasets and detection methods based on convolutional and transformer architectures (Verdoliva et al., 2019; Li et al., 2020).

Despite these parallel developments, current literature remains fragmented. Few studies systematically integrate technical detection methods with social, ethical, and policy perspectives. This review addresses that gap by synthesizing interdisciplinary research to provide a unified understanding of deepfake creation, detection, and governance. Specifically, it seeks to:

- Identify dominant technical and social approaches of deepfake generation and detection.
- Evaluate how explainable AI and multimodal architectures enhance detection robustness.
- Examine how regulatory and ethical frameworks can inform the design of responsible detection systems.

By combining insights from computer vision, natural language processing, explainable AI, and social science, this review provides a cross-disciplinary taxonomy of deepfake research and outlines a conceptual framework for integrating detection, explainability, and governance. Through this synthesis, it aims to support the development of transparent, ethical, and technically resilient AI-based systems for mitigating the harms of synthetic media.

In line with contemporary academic consensus, this review avoids treating “fake news” as a standalone analytical category due to its politicized and ambiguous usage. Rather, the terms misinformation, disinformation, and AI-created misleading content are employed to differentiate unintentional errors, intentional distortion, and artificial media products, respectively. This terminological precision enables clearer alignment between technical detection methods, social impact studies, and regulatory frameworks.

## 2 Methodology

### 2.1 Review protocol and literature selection

This study adopted a systematic review approach to synthesize current research on deepfakes, generative Artificial Intelligence (AI), and misinformation. The review followed the Preferred Reporting Items for Systematic Reviews and Meta-Analyses (PRISMA) guidelines to ensure methodological transparency, replicability, and rigor. The process involved six key stages: (i) defining the research scope and questions, (ii) developing search strategies and selecting databases, (iii) applying inclusion and exclusion criteria, (iv) screening and extracting relevant data, (v) assessing methodological quality, and (vi) synthesizing and interpreting the findings.

The scope of this review covered the period 2014 to 2025, reflecting the evolution of deepfake research from its early technical demonstrations to contemporary multimodal and regulatory developments. Both qualitative and quantitative evidence were considered to capture the interdisciplinary nature of deepfake research across technical, social, and policy domains.

### 2.2 Search strategy and databases

A comprehensive multi-database search strategy was designed to capture the full breadth of deepfake-related research, including studies on generation, detection, misinformation, and regulation. The Boolean search string used (Scopus format) was:

(“deepfake*” OR “fake news” OR “synthetic media” OR “AI-generated content” OR “misinformation” OR “disinformation”) AND (“artificial intelligence” OR “machine learning” OR “deep learning” OR “neural network*” OR “natural language processing” OR “computer vision”)

From an initial pool of 120 database records, complemented by backward and forward citation chaining, this query was executed and adapted for each database, including IEEE Xplore, ACM Digital Library, Scopus, SpringerLink, and Web of Science. To ensure inclusion of the most recent advances, arXiv preprints were also screened, recognizing that peer review often lags rapid developments in generative AI.

Preprints were included only when they were later cited in peer-reviewed venues or associated with publicly recognized datasets to mitigate quality concerns.

For regulatory and governance perspectives, official EU repositories and government sites were reviewed to obtain key policy texts such as the EU Digital Services Act (European Parliament and Council of the European Union (EU DSA), 2022) and the EU AI Act (European Parliament and Council of the European Union (EU AI Act), 2024). Reference lists of selected articles were also examined through backward and forward citation chasing to identify additional relevant studies not captured by the initial search.

arXiv preprints were included selectively due to the rapid pace of advances in generative AI and were cross-validated based on subsequent peer-reviewed adoption, dataset impact, or citation prominence.

### 2.3 Inclusion and exclusion criteria

Inclusion criteria:

Studies were included if they met the following criteria:

- Published between 2014 and 2025;
- Peer-reviewed journal articles, conference papers, or preprints focusing on deepfake generation, detection, or governance;
- Addressed technical, social, or policy/regulatory dimensions of deepfakes;
- Investigated text, audio, image, or video modalities related to synthetic media;
- Written in English and available in full-text form.

Exclusion criteria:

- Studies published in languages other than English;
- Duplicate records across multiple databases;
- Non-peer-reviewed sources such as blog posts, news articles, and unverified reports (except official institutional or legal documents);
- Studies focusing solely on unrelated AI applications (e.g., generative art) without relevance to misinformation or deepfake detection;
- Theoretical discussions lacking empirical or methodological depth.

These criteria ensured that the final selection consisted of studies with direct relevance, methodological rigor, and conceptual clarity.

### 2.4 Search strategy

The search strategy combined structured database keyword searches with both backward citations chasing (examining the references of included studies) and forward citation chasing (tracking newer works that cited them). Initial queries employed broad terms such as *deepfake detection*, *generative adversarial networks (GANs)*, *synthetic media*, *misinformation*, and *fake news*. Throughout the review, the strategy was refined repeatedly to incorporate new methods and ideas. Other keywords comprised transformers, Vision Transformer (ViT), CLIP, voice generation, multimodal recognition, Xplainable AI (XAI), retrieval-augmented production (RAG), bias, equity, governance, and regulation. Boolean operators (e.g., “deepfake AND identification”, “synthetic media AND false information”, “transformer OR CLIP AND deepfake”) were employed to enhance recall and precision. This iterative approach ensured that both foundational studies and the most recent developments in technical, social, and regulatory domains were systematically captured.

### 2.5 Screening process

The screening process followed a three-stage procedure to ensure systematic inclusion of high-quality and relevant studies:

1. Title and abstract screening: all retrieved records were reviewed to exclude irrelevant topics, such as unrelated computer vision tasks or non-AI media analyses.
2. Full-text review: remaining articles were evaluated for methodological soundness, empirical contribution, and relevance to the research objectives.
3. Thematic categorization: eligible studies were coded into thematic clusters representing:

(i) Dataset creation and benchmark development.
(ii) Detection models and architectures.
(iii) Explainability and adversarial robustness.
(iv) Social trust and misinformation.
(v) Policy and regulatory frameworks.

At the full-text screening stage, studies were excluded for the following predefined reasons:

Reason 1: Primary focus on AI applications unrelated to misinformation or deceptive synthetic media (*n* = 6).

Reason 2: Lack of empirical evaluation, methodological transparency, or reproducible analysis (*n* = 5).

Reason 3: Conceptual or opinion-based papers without sufficient analytical depth or evidence synthesis (*n* = 7).

#### 2.5.1 Data extraction process

Data extraction was conducted using a structured data extraction form developed in Microsoft Excel. Two reviewers independently extracted data to minimize bias, with discrepancies resolved through discussion and consensus.

Extracted fields included:

- Author(s), year, and publication type.
- Research domain and methodology.
- AI model or framework (e.g., GAN, transformer, CLIP).
- Dataset characteristics and evaluation metrics.
- Key findings and thematic relevance.

This approach ensured consistency and completeness in capturing both technical and contextual details.

Inter-rater reliability between reviewers was assessed using Cohen’s kappa coefficient (*κ*), which indicated strong agreement during the screening and data extraction phases.

### 2.6 Number of studies included

The systematic review included a total of 34 studies published between 2014 and 2025, capturing both the historical development and the latest advances in deepfake research. About 18 of them examined deepfake production, detection methods, and model architectures with an emphasis on computer vision and technical approaches. About eight studies from the social sciences looked at how deepfakes affect user behaviour, media trust, and disinformation in society. The other 8 studies focused on policy and regulatory frameworks, emphasizing ethical issues, governance strategies, and legal actions. Research was chosen for its capability to offer a broad viewpoint across various media formats text, images, video, and audio and for its role in enhancing knowledge of the technical, social, and policy aspects of deepfakes. This selection ensures a holistic view of the field, combining insights into both innovation and the societal challenges posed by synthetic media.

Although the ultimate set of 34 studies may seem narrow compared to the vastness of generative AI research, this demonstrates the careful use of stringent inclusion standards that prioritize interdisciplinary significance, methodological soundness, and clear involvement with detection, social consequences, or governance. This trade-off prioritizes analytical depth and coherence over exhaustive coverage and is acknowledged as a limitation of the review.

### 2.7 Quality assessment

The methodological quality and potential risk of bias for each study were evaluated using an adapted version of the Critical Appraisal Skills Program (CASP) checklist. Technical studies were assessed based on criteria such as dataset transparency, model reproducibility, and evaluation robustness, while social science and policy studies were reviewed for methodological clarity, validity of interpretation, and evidence linkage. Each study received a quality rating (high, moderate, or low) based on these criteria, which informed the weight given during synthesis of the 34 studies evaluated, 21 were rated high quality, 9 moderate, and 4 low according to the adapted CASP checklist.

### 2.8 Rationale for selection

**Figure 1:**
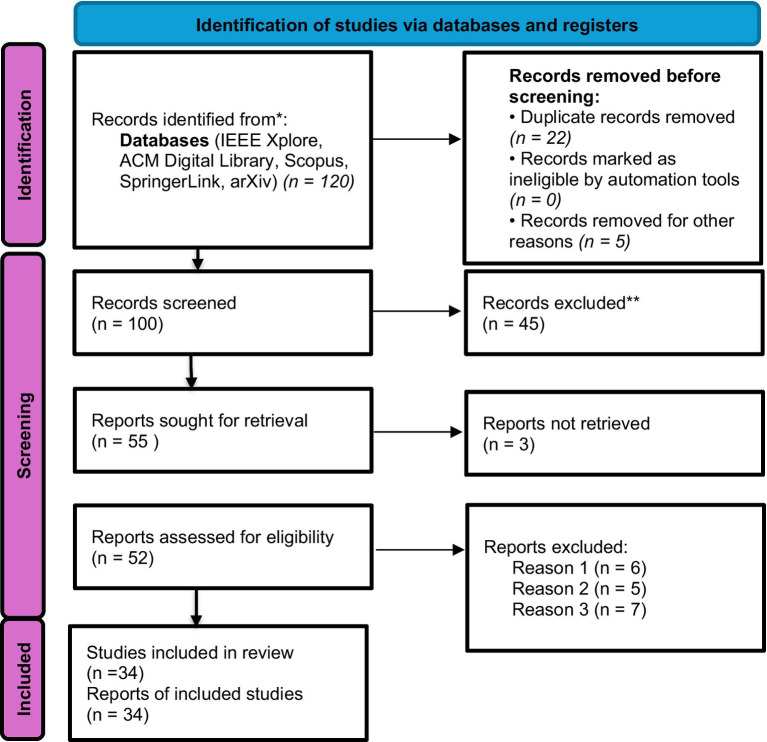
PRISMA flowchart.

The studies included in this review were selected for several key reasons. They first offer a historical basis for comprehending the evolution of generative AI and the rise of misinformation by illustrating the progression of deepfakes from early technical demos to highly advanced synthetic media. Second, they showcase state-of-the-art technological detection techniques, such as multimodal analysis, explainable AI, and machine learning model advancements. Third, the research investigates sociological, ethical, and legal aspects, analysing how deepfakes influence media credibility, public opinion, privacy, and regulatory systems. Finally, the selection deliberately encompasses a range of media modalities text, images, video, and audio to ensure a comprehensive understanding of both the technical challenges and societal implications of deepfakes (see Figure 1).

## 3 Thematic review of literature

### 3.1 Overview of thematic analysis

A thematic review approach was used to organize and interpret the selected studies according to recurring patterns, concepts, and research priorities. Finding commonly discussed subjects, assembling related concepts into clusters, and honing these clusters into cohesive themes were the steps in the analysis process. This approach facilitated the emergence of both chronological and conceptual links, underscoring the progression of research on deepfakes and synthetic media from initial technical trials to wider societal and policy-focused conversations.

From this analysis, four overarching themes were identified:

- Evolution of Deepfake Technologies and Detection Research;
- Technical developments in deepfake generation and detection methods;
- Explainability, robustness, and evaluation challenges in AI-based detection;
- Social and psychological impacts, including misinformation, media trust, and user behaviour;
- Governance, ethics, and policy frameworks addressing regulation and accountability.

### 3.2 Evolution of deepfake technologies and detection research

The trajectory of deepfake development mirrors broader advances in generative AI. Early methods, notably Generative Adversarial Networks (GANs) (Goodfellow et al., 2014) established the adversarial paradigm that underpins synthetic image and video generation. Subsequent variants such as StyleGAN2/3 (Lehtinen & Aila NVIDIA) dramatically improved realism and controllability. Meanwhile, transformer architectures (Vaswani et al., 2017) and large-scale language models (Brown et al., 2020) expanded synthetic generation to text and audio, creating a multimodal threat landscape.

These architectural advances not only enabled high-fidelity media synthesis but also reshaped detection research. As deepfakes grew more realistic, early CNN-based detectors emphasizing pixel-level inconsistencies gave way to transformer and CLIP-based frameworks capable of modelling contextual and semantic relationships. This shift reflects an evolution from surface-level artifact detection toward cross-modal, meaning-aware analysis, mirroring the progression of misinformation itself from isolated falsifications to integrated multimodal narratives.

This architectural transition reflects more than incremental performance improvement. Detectors based on CNNs chiefly leverage low-level visual artifacts, which modern generative models increasingly reduce. Transformer and CLIP-based approaches instead model long-range spatial, temporal, and semantic dependencies, enabling improved robustness to compression and post-processing. Nonetheless, these benefits come with trade-offs in computational expense, data reliance, and interpretability. The literature rarely addresses how such models can be deployed in real-time or resource-constrained environments, revealing a gap between benchmark success and operational feasibility (see Table 1).

**Table 1:** Summary of emerging themes in the current state of knowledge.

Theme	Focus area	Key findings	Representative studies/datasets
1. Advances in datasets and detection models	Development of datasets and model architectures for detection	Expansion from CNNs to Transformer-based and CLIP-integrated models; improved generalization via multimodal benchmarks	FaceForensics++, DFDC, Celeb-DF, FakeVoices, WaveFake, Deepfake-Eval-2024
2. Explainability and adversarial robustness	Interpretability and resilience of detection systems	Use of Grad-CAM and similar methods; emerging risks from explainability-based attacks	Selvaraju et al. (2017)
3. Social, ethical, and policy responses	Societal impacts and governance frameworks	Rising misinformation, erosion of trust, regulatory responses via EU AI and DSA Acts	Idiongo (2024), National Sexual Violence Resource Center (2024), and European Parliament and Council of the European Union (EU DSA) (2022, 2024)

### 3.3 Comparative analysis

To synthesize and contrast the findings across the selected studies, a comparative analysis was performed focusing on methodological design, evaluation performance, and disciplinary orientation. To find similarities, contrasts, and latest trends, this analysis combines data from computer vision, multimodal AI, social science, and policy research.

A comparative analysis identified three primary methodological clusters CNN-based, Transformer-based, and CLIP-based/multimodal frameworks each presenting unique advantages and disadvantages. CNN architectures (e.g., Section 3: Thematic Review/Detection Models, EfficientNet) exhibit high accuracy on established benchmarks such as FaceForensics++ and DFDC but demonstrate limited generalization to unseen datasets or manipulation techniques. Although they require more data and have greater computing costs, Vision Transformers (ViT, Swin-T) and hybrid models provide better contextual modeling and modest improvements in cross-dataset generalization. CLIP-based and multimodal methods utilize vision-language pretraining to enable zero-shot and few-shot detection, indicating scalability potential across modalities, yet they continue to be vulnerable to adversarial perturbations and domain shifts.

Recent research has delved deeper into multimodal fusion and extensive pretraining techniques to enhance generalization and resilience in deepfake detection, especially in cross-dataset and real-world scenarios (Chen et al., 2024a; Chen et al., 2024b).

Foundational deep learning architectures underpinning contemporary detection systems include Xception networks (Chollet, 2017), transformer-based language models such as BERT (Devlin et al., 2019), and generative adversarial networks including StyleGAN (Karras et al., 2021). These architectures have directly impacted the generation pipelines for deepfakes and the detection methods assessed in benchmark datasets like FaceForensics++ (Dolhansky et al., 2019).

From a disciplinary standpoint, methodological research use diverges:

- Computer vision studies prioritize quantitative metrics such as AUC, accuracy, and F1-score, emphasizing model robustness and scalability.
- Social science research focuses on user perception, misinformation spread, and trust restoration, prioritizing ecological validity over computational precision.
- Policy and governance studies emphasize legal accountability, transparency mechanisms, and platform obligations, focusing less on algorithms and more on institutional enforcement.

#### 3.3.1 Quantitative overview of methodological clusters

Among the 34 studies analysed, approximately 59% (*n* = 20) employed CNN-based detection models, 26% (*n* = 9) used transformer or hybrid architectures, and 15% (*n* = 5) adopted multimodal or CLIP-based approaches. Social science and policy-focused studies comprised ~30% of the total dataset, underscoring the growing interdisciplinary scope of deepfake research.

The predominance of CNN-based approaches (59%) reflects both historical inertia and dataset availability, as many widely used benchmarks were designed to expose CNN-detectable artifacts. While this has accelerated short-term performance gains, it may also constrain innovation by incentivizing dataset-specific optimization rather than real-world generalization. This methodological concentration highlights the need for evaluation protocols that reward robustness, multimodal reasoning, and cross-domain adaptability (see Table 2).

**Table 2:** Comparative summary of detection approaches and research perspectives.

Approach domain	Representative studies	Core techniques	Strengths	Limitations	Evaluation focus metrics
CNN-based detection	Li et al., 2020 and Verdoliva et al. (2019)	XceptionNet, EfficientNet, ResNet	High benchmark accuracy, efficient training	Poor cross-dataset generalization; sensitive to new manipulations	AUC, F1-score, accuracy
Transformer-based models	Chen et al. (2024a, 2024b) and Wang et al. (2024)	ViT, Swin Transformer, hybrid CNN-Transformer	Captures long-range dependencies, strong contextual modelling	Data-hungry, computationally expensive	Accuracy, cross-dataset robustness
CLIP multimodal approaches	Yermakov et al. (2025) and Brown et al., 2025	Vision–language pretraining, frozen CLIP features	Zero-shot and multimodal generalization; scalable	Adversarial vulnerability, distribution shift	Zero-shot accuracy, multimodal retrieval performance
Explainable & robust AI	Kozik et al. (2024) and Selvaraju et al. (2017)	Grad-CAM, feature attribution, robustness testing	Transparency, user interpretability	Risk of adversarial exploitation	Interpretability quality, robustness metrics
Social behavioural studies	Idiongo (2024) and Zhou et al. (2021)	Surveys, experiments, discourse analysis	Insights into trust, misinformation spread	Lack of quantitative precision	User trust, perception, misinformation spread
Policy & regulatory studies	European Parliament and Council of the European Union (EU DSA) (2022) and European Parliament and Council of the European Union (EU AI Act) (2024)	Legislative review, policy analysis	Governance, accountability frameworks	Limited enforcement mechanisms, lag behind tech	Policy compliance, transparency obligations

#### 3.3.2 Interpretation and implications

The comparative synthesis highlights that while technical advancements in AI detection (e.g., transformers, CLIP) show promise, cross-domain generalization and ethical integration remain unresolved. Complementary insights on trust and governance are offered by social and policy studies, indicating the necessity of interdisciplinary frameworks that combine societal resilience, regulatory oversight, and technical detection accuracy. These comparative insights form the foundation for the conceptual framework presented in the next section, integrating strengths across disciplines to advance comprehensive deepfake mitigation strategies.

### 3.4 Conceptual framework

**Figure 2:**
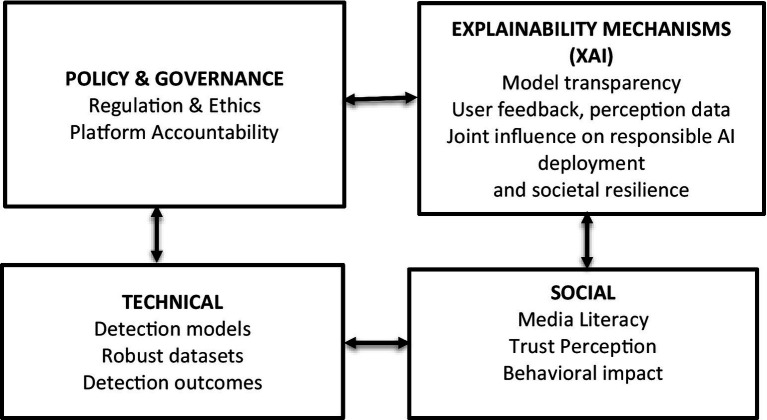
Conceptual framework.

The framework integrates technical, social, and governance dimensions through feedback loops that connect model transparency, user trust, and regulatory compliance to build accountable and resilient AI detection systems (see Figure 2).

The framework is organized in a three-tier system:

1. Technical layer – encompassing detection models, detection outcomes and robust datasets;
2. Social layer – focusing on media literacy, trust perception, and behavioural impact;
3. Policy & governance layer – covering regulation, ethics, and platform accountability;
4. Explainability mechanisms – dealing with model transparency, user feedback, perception data, joint influence on responsible AI deployment and societal resilience.

These components are interconnected through bidirectional feedback loops. Technical detection systems generate transparent outputs that inform social understanding and policy decisions, while governance structures provide ethical and compliance feedback to guide model development and user interaction.

This framework extends prior conceptual models (Mirsky and Lee, 2021; Zhou et al., 2019) by explicitly incorporating explainability and feedback dynamics that connect machine learning performance with human trust and policy accountability. Unlike earlier models focusing solely on detection or social impacts, this integrated design emphasizes *mutual reinforcement* between technology, transparency, and governance to create a resilient AI ecosystem for mitigating deepfake threats.

#### 3.4.1 Operationalization of the conceptual framework

The framework is designed to function as an operational model rather than a static taxonomy. In the technical layer, detection models analyze multimodal content and produce classification results along with explainability indicators like confidence scores or attention maps. These outputs directly influence the social layer by shaping moderation decisions, media literacy interventions, and user trust calibration.

User engagement, such as requests, modifications, and sharing actions, generates feedback data that returns to the technical layer, guiding dataset improvement, bias assessment, and model updates. Simultaneously, the policy and governance layer constrains system behaviour through transparency obligations, accountability requirements, and platform-level enforcement mechanisms, which shape both technical design and social response strategies.

In practical scenarios such as election integrity or non-consensual deepfake mitigation, this closed-loop structure enables continuous alignment between detection accuracy, societal trust, and regulatory compliance.

In contrast to earlier multi-layer frameworks that mainly classify stakeholders or research areas, this framework specifically represents feedback loops among technical outputs, human understanding, and governance limitations. This enables evaluation of how design choices in detection and explainability propagate through social trust and regulatory accountability, making the framework applicable to system design, policy assessment, and interdisciplinary research planning.

### 3.5 Knowledge gaps

Despite significant advances in deepfake detection, several key gaps and contentious issues remain. Generalization is a major challenge, as many detectors perform well on benchmark datasets but struggle to maintain accuracy across new or unseen datasets, limiting their real-world applicability. Multimodal deepfakes, which combine video, audio, and text, are understudied compared to single-modality cases, leaving detection methods less prepared for increasingly sophisticated manipulations. The balance between explainability and security also presents a dilemma: while explainable AI tools improve transparency, they can potentially be exploited by adversaries to bypass detection. Additionally, ethical and legal protections remain insufficient, and harmful such as non-consensual deepfake pornography and political disinformation highlight gaps in current laws and enforcement. Finally, the lack of standardized, adversarial resilient evaluation benchmarks complicates the comparison of detection methods across studies, making it difficult to assess progress and deploy robust solutions in practice.

## 4 Critical discussion

The literature reveals a field advancing rapidly on the technical front while struggling to ensure adequate societal protection. Although detection models have improved significantly, the adversarial dynamics of the problem mean that innovations in synthesis frequently outpace detection capabilities. This ongoing arms race is intensified by dataset biases, inconsistent reporting standards, and fragmented evaluation practices.

In addition, the interdisciplinary gaps persist until today. Social scientists extensively document the social and behavioural consequences of misinformation but often neglect the technical constraints of detection systems. Conversely, many computer vision studies validate models using synthetic benchmarks without evaluating real-world or societal implications. Regulatory initiatives such as the EU AI Act and Digital Services Act introduce frameworks for accountability, yet translating these high-level principles into enforceable technical standards and platform practices remains an open challenge.

Previous surveys and forensic studies established the foundation for contemporary detection research by systematizing manipulation categories and assessment methods (Verdoliva et al., 2019; Zhou et al., 2021).

Deployment challenges also include scalability, privacy protection when handling sensitive data, and the ethical management of false positives that could affect legitimate content. These challenges directly relate to the conceptual framework proposed in this review: weaknesses in the *technical domain* (e.g., bias, overfitting) affect *social trust and media literacy*, while insufficient *policy enforcement* weakens governance feedback loops. A more integrated research agenda that unites robust technical evaluation, human-centred design, and co-developed regulatory mechanisms is necessary to ensure that progress in detection contributes meaningfully to societal protection.

Recent regulatory initiatives further underscore the need for accountable AI-driven detection systems. The Digital Services Act (European Parliament and Council of the European Union (EU DSA), 2022) of the European Union establishes requirements for platform transparency and risk reduction concerning online misinformation, while the proposed European Union Artificial Intelligence Act (European Parliament and Council of the European Union (EU AI Act), 2024) classifies certain AI-driven content moderation and synthetic media systems as high-risk, imposing requirements for explainability, documentation, and human oversight.

## 5 Future directions

Future research on deepfake detection must move beyond narrow, dataset-driven evaluations toward holistic, context-aware, and ethically aligned systems. Guided by the conceptual framework, four thematic directions namely technical, methodological, ethical, and policy/governance are proposed to structure future development.

### 5.1 Technical directions

Future detection models should reflect the complexity of multimodal manipulations and evolving threat landscapes:

- Multimodal Benchmark Development: Construct datasets that jointly assess image, video, audio, and text manipulations to capture cross-modal deepfake narratives.
- Cross-Domain Generalization: Mitigate overfitting through domain adaptation, self-supervised learning, and transfer learning to enhance robustness across datasets and manipulation types.
- Retrieval-Augmented Detection: Incorporate external evidence (e.g., verified media or fact databases) into detection reasoning for improved factual grounding and reliability.
- Explainability and Robustness: Advance interpretable AI methods (e.g., saliency maps, attention visualizations) that clarify decisions and resist adversarial exploitation.

### 5.2 Methodological directions

- Adversarial-Resilient Evaluation Protocols: Establish standardized benchmarks that test model performance under realistic, adversarial, and cross-cultural conditions.
- Human–AI Collaboration: Design hybrid systems where explainable AI tools assist human reviewers, journalists, and policymakers in verifying authenticity and contextual accuracy.
- Longitudinal and Cross-Platform Studies: Examine how detection systems perform across time and social media ecosystems to measure real-world efficacy and adaptation.

### 5.3 Ethical directions

- Data Privacy and Consent: Ensure deepfake datasets use consensual, privacy-preserving data to avoid reinforcing exploitation or harm.
- Bias and Fairness Auditing: Implement fairness checks and demographic audits in training datasets to prevent disproportionate impacts on marginalized groups.
- Transparency and Accountability: Promote open reporting of model architectures, performance metrics, and limitations to support reproducibility and ethical oversight.

### 5.4 Policy and governance directions

- Regulatory alignment: translate policy instruments such as the *EU AI Act* and *Digital Services Act* into technical compliance standards, platform-level transparency, and auditable mechanisms.
- Global governance frameworks: encourage international cooperation to develop harmonized policies for detecting and labelling synthetic media.
- Public education and media literacy: foster interdisciplinary collaboration between AI developers, educators, and communication experts to build societal resilience against misinformation.

#### 5.4.1 Practical implications

For researchers, this framework encourages the integration of technical and social dimensions, ensuring that advances in model accuracy are matched by attention to transparency and fairness. Policy makers can use it to align AI regulation with technical feasibility, creating compliance standards that are both enforceable and adaptable. Media platforms can operationalize these insights by embedding explainable detection tools within content moderation pipelines. Collectively, these directions emphasize that the sustainability of deepfake detection depends on continuous interaction between innovation, ethics, and governance.

## 6 Conclusion

This review synthesizes technical, social, and policy literature on deepfakes and detection. While there has been commendable progress in detection techniques and benchmark creation, significant challenges remain in generalization, multimodal detection, ethical safeguards, and policy enforcement. Many current models achieve strong results on specific datasets, yet their reliability weakens in real-world contexts where manipulations are more diverse and adversarial adaptive. Multimodal deepfakes, which combine video, audio, and textual fabrications, further complicate detection by exploiting gaps between unimodal approaches.

Beyond technical hurdles, unresolved ethical and legal questions persist. Non-consensual sexual deepfakes and political disinformation highlight the potential for severe personal and societal harm, underscoring the urgency of legal protections, content moderation frameworks, and victim support mechanisms. Policies such as the EU AI Act and Digital Services Act provide valuable blueprints, but enforcement depends on effective translation into technical standards and platform practices. At the same time, overzealous regulation risks constraining legitimate research and creative uses of generative technologies, demanding careful balance.

Addressing these challenges requires more than technical innovation; it demands sustained interdisciplinary collaboration. Engineers, social scientists, policymakers, and ethicists must co-design solutions that are simultaneously robust, explainable, and aligned with democratic values. Evaluation standards should evolve toward adversarial resilient benchmarks that mirror deployment conditions, while explainability tools must empower human reviewers without exposing new attack surfaces.

The promise and perils of generative AI demand coordinated responses that protect individuals and societies while enabling beneficial innovation. If pursued with transparency, inclusivity, and foresight, the next generation of deepfake detection systems and governance structures can mitigate harms while fostering responsible use of synthetic media in education, entertainment, accessibility, and beyond. The trajectory of this field will depend on whether the global community can act not only to keep pace with technological advances, but also to shape them in the service of public trust and social good.

## Data Availability

Publicly available datasets were analyzed in this study. This data can be found here: FakeNewsNet for text, FaceForensics++ for video.
